# Examining the influence of scarcity, weather, and vacation on physicians' performance, mortality, and costs

**DOI:** 10.7150/ijms.124438

**Published:** 2026-06-04

**Authors:** Óscar Gasulla, Antonio Sarría-Santamera, Ferran A Mazaira-Font, Cielo García-Montero, Oscar Fraile-Martinez, Diego Cantalapiedra, Manuel F Carrillo-Rodríguez, Belen Gómez-Valcárcel, Miguel Á Ortega, Melchor Álvarez-Mon, Angel Asúnsolo

**Affiliations:** 1 Hospital Universitari de Bellvitge, Universitat de Barcelona, L'Hospitalet de Llobregat, 08907 Barcelona, Spain.; 2 Department of Surgery, Medical and Social Sciences, Faculty of Medicine and Health Sciences, University of Alcalá, 28801 Alcalá de Henares, Spain.; 3 Department of Biomedical Sciences, Nazarbayev University School of Medicine, Astana 010000, Kazakhstan.; 4 Departament d'Econometria, Estadística i Economia Aplicada, Universitat de Barcelona, 08907 Barcelona, Spain.; 5 Department of Medicine and Medical Specialities, Faculty of Medicine and Health Sciences, University of Alcalá, 28801 Alcalá de Henares, Spain.; 6 Ramón y Cajal Institute of Sanitary Research (IRYCIS), 28034 Madrid, Spain.; 7 Faculty of Medicine, Universidad Europea of Madrid, 28670 Madrid, Spain.; 8 Networking Research Center for Liver and Digestive Diseases (CIBEREHD), Immune System Diseases-Rheumatology and Internal Medicine Department, Príncipe de Asturias University, 28806 Alcalá de Henares, Spain.; 9 Department of Epidemiology and Biostatistics, Graduate School of Public Health and Health Policy, University of New York, New York, NY 10027, USA.

**Keywords:** focus, medical performance, scarcity, percutaneous coronary intervention (PCI), coronary artery bypass grafting (CABG)

## Abstract

Healthcare professionals are exposed to different factors that may affect their ability for decision-making. The socioeconomic and health system impacts of scarcity and focus on workflow serve as a challenge to improve resource management and maintain focus. In this study, we analyze a dataset of patients who underwent percutaneous coronary intervention (PCI) and coronary artery bypass grafting (CABG) in Spain between 2010 to 2012, and 2016 to 2019, being either programmed or urgent interventions. We associated the results with a dataset from the Spanish Meteorological Agency to assess if the weather could affect the focus of physicians and the results of mortality after the intervention. We also considered if there was a relationship when intervention was made on vacation days or weekends. The results show that scarcity, studied as urgent interventions, and lack of focus, such as vacation days, increase mortality and costs of surgical interventions. Rainy days were found to be a factor that increases focus. Eventually, these results may allow proposing strategies that benefit physicians' performance, resource management, and treatment options, besides the psychological impact on patients, or even avoiding medical errors. Moreover, the same analysis could be applied to other jobs to increase productivity in the economy.

## 1. Introduction

Over the past decade, academic interest has increased in understanding how scarcity and cognitive focus influence decision-making and performance in medical care. These two concepts are central to cognitive processing, therefore, having an impact on healthcare delivery.

Scarcity refers to a condition in which available resources are insufficient to meet existing demands in an optimal way 1. These resources may include time, financial means, personnel, equipment, or institutional capacity. Research on scarcity has largely emerged from economics and public policy, particularly following the 2008 financial crisis and the COVID-19 pandemic in 2020, which exposed widespread limitations in healthcare and social systems 3. When individuals or organizations operate under scarcity, they must continuously manage urgent resource constraints, which reduces the cognitive resources available for decision-making and action 4. Because human cognitive capacity is limited, persistent concerns about time, money, or workload can impair judgment and performance.

Cognitive focus refers to the brain's ability to direct attention toward a specific objective through information gathering, retention, and sustained mental effort over time 2. Focus is essential for higher-order cognitive functions, including reasoning, planning, and complex decision-making 5,6. Time pressure and competing demands typically weaken concentration and reduce performance across a wide range of tasks, from simple calculations to highly technical activities such as performing surgery. In healthcare settings, diminished focus increases the risk of errors and reduce the quality of clinical decisions.

In medicine and health economics, previous research has examined how limited resources and restricted accessibility influence prioritization and clinical outcomes. Studies have shown that organizational focus -at the hospital, unit, and process-flow level- is associated with improved performance 7-9. Better performance -in terms of quality and efficiency- has also been linked to focus and professional experience 8. These findings suggest that both focus and scarcity may play an important role in medical performance.

Based on a database of percutaneous coronary intervention (PCI) and coronary artery bypass grafting (CABG) procedures and a dataset from the Spanish Meteorological Agency, this study investigates the existence of correlation between mortality and costs on one hand, and focus and scarcity on the other; being focus associated with less mortality and cost, and scarcity with more mortality and costs.

## 2. Patients and Methods

The present study was designed as an observational, analytical, retrospective cohort study. This study was carried out with the database from the Public Health Ministry of Spain, containing all the hospital discharges from PCI and CABG procedures in Spain, between the years 2010 to 2012, and from 2016 to 2019. A total number of 404.918 hospital discharges are registered.

For each hospital discharge, three types of information relevant for our study are provided: socio-demographical, medical, and financial. Socio-demographical data includes age, gender, and Spanish region of origin. Medical data describes the type of intervention (PCI or CABG), its urgency, the date of the intervention, codified diseases the patient was diagnosed (CIE-9-MC for years 2010 to 2012, and CIE-10-ES afterwards), and whether the result of the hospital discharge was an exitus or not. We consider the main comorbidities associated with procedure selection and mortality risk in CABG and PCI interventions: diabetes mellitus, kidney diseases and cardiac insufficiency 10-13. Finally, in terms of financial information, the associated cost of the procedure is provided.

In order to analyze the impact of scarcity and focus on medical performance, we assigned these effects with three variables. To capture scarcity, we consider the dummy *Urgent*, that equals 1 if the intervention was not programmed. It is important to note that the Urgent variable may capture not only a scarcity effect, but also a deterioration in the patient's condition that is not explained by the other available variables. To account for focus, we considerate two variables. The first one, named *Vacations*, equals 1 if the date of the intervention was Friday, Saturday, or a bank holiday in Spain or the Region where the intervention took place. The second variable, named *RainyDay*, equals 1 if the date of the intervention was a rainy day (> 2 l/m2) at the municipality where the intervention took place. The data was obtained from the *Agencia Española de Meteorología* (Spanish Meteorological Agency).

Table 1 shows a description of the demographic, medical and financial data of the hospital discharges involved in the study, by procedure. We also include the variables related with scarcity and focus.

To test the aforementioned effects, we use multivariate models. We build two different models. The first model is a logistic regression whose target is the medical outcome of the procedure and equals 1 if the outcome was an exitus. The second is a Gaussian regression whose target is the cost of the procedure. In both cases, we test one at a time whether the effect of scarcity variables or focus variables is significant, adjusted by demographic and medical information. That is, we test whether focus or scarcity alter the outcome or the cost of the procedure adjusting for patient variability. We build separate models for PCI and CABG.

## 3. Results

We report the estimated effects of the Focus and Scarcity variables in Table 2. Since the parameters of a logistic model cannot be directly interpreted in terms of the variables' marginal impact, but rather through the logistic transformation, we compute the estimated effect on mortality based on the model's predictions under scenarios in which the Focus and Scarcity variables are set to 1 and to 0, respectively. As it can be seen in Table 2, urgent procedures have higher mortality rates than scheduled procedures, both in CABG and PCI. Namely, mortality risk adjusted by comorbidities, sex, and gender, increases by 1,5 and 1,7 percentage points, respectively. Regarding the variables related with focus, both have the expected sign.

On the one hand, *Vacations* decreases focus, and therefore, increases mortality risk. The increase is significant for PCI but is not significant in the case of CABG. On the other hand, *Rainy day* increase focus, and as a result, decreases mortality rate in the two procedures. The effect is highly significant for CABG, but low significant for PCI. The impact on mortality of the focus effects is one order of magnitude lower than scarcity effects. This difference may partly arise because the Urgent variable captures both scarcity effects and unobserved deterioration in the patient's condition.

Table 3 shows the impact of scarcity and focus in terms of cost. As in the case of the impact on mortality, the effect of scarcity (urgent procedures) is highly significant in PCI and CABG and has the expected sign. The higher the scarcity, the higher the cost due to the lack of time to plan in advanced, and, potentially, the higher complexity to deal with the procedure in such context. Recall that the effect in cost is estimated adjusting by comorbidities and demographic data. Hence, it is already adjusted by the available information of patient's complexity. Focus variables have all the expected effect. Higher focus (*Rainy day*) reduces costs, while lower focus (*Vacations*) increases costs, other things equal. As with the effect on mortality, focus effects on costs are one order of magnitude (or even two) lower than those of scarcity.

To summarize, our estimates show that scarcity and focus have significant effects in surgery performance, both in terms of mortality and costs. Scarcity and lack of focus increases mortality and costs, while strong focus reduces both.

## 4. Discussion

In summary, scarcity, studied as urgent interventions, and lack of focus, as vacation days, increase mortality and costs of surgical interventions, while strong focus reduces both, although the overall positive effects of focus have less impact when compared with the negative effects of scarcity.

The results of this study are in line with others found in the scientific literature 7-9. In terms of scarcity, due to COVID-19 pandemic, in another study they made an experiment assessing medical decisions with different kind of patients. Such financial adjustments most likely have an impact on how healthcare is provided. Therefore, they found that when physicians are under more severe financial pressure, they offer fewer services. This is, as physician expenses increase, average patient benefits drop 14.

Therefore, policies that seek to have professionals in the lowest condition of scarcity are beneficial for our healthcare system. As an example, the shift from a traditional schedule with extended (24 hours or more) to an intervention schedule that eliminates extended work shifts and reduced the number of hours worked per week has been found to enhance physicians' performance 15. Finding ways to increase the focus of medical professionals also should turn into better outcomes as well.

Scarcity and focus could have an impact on performance in two different ways. They can alter the medical outcome of the procedure, by affecting the mortality rate, or can alter its costs. It may be noticed that either if they increase mortality rate while keeping costs unaffected, or increase costs while keeping mortality unaffected, they would be reducing performance.

As focus proxies we used festivity days as a factor that reduces focus and rainy days as a factor that increases focus in medical performance. The results of this paper are consistent with many studies that have found a reduction of physician's performance when working overnight, on urgent interventions and on duty calls, where a situation of scarcity lead the professional to more medical errors 13,16,17. As reported in literature, we have also found better performance during rainy days 18. Economics research explains that rainy days are linked to less leisure time enjoyment, which increases wages and adds to the number of hours worked. They noticed that men move from leisure to work for an average of 30 minutes on rainy days 19.

We could not find other studies that describe a reduction in productivity on Fridays in medical field but there are other general studies concerning productivity variance across days of the week. Long was discussed about related consequences like illness, absence or error rates 20. More recently, some data mining tools have been applied massively to business works, evaluating the productivity of their employees. These studies found that Fridays had a 25% lower rate of production than other days of the week 21. That could be explained by workers being more distracted by the incoming weekend.

Conversely, stress, lack of sleep, loneliness, and inactivity all affect executive functions, hence the significance of social, emotional, and physical health for cognitive health is highlighted 5. These results show once again the importance of the motivational and concentration environment in jobs as complex as medicine, especially in some specialties such as surgery 22. Further research could be conducted to assess the impact of scarcity and focus on other medical procedures.

## Figures and Tables

**Table 1:** Description of the data

	PCI	CABG
**Demographics**		
Age	66,0	67,3
		
Women	23,4%	20,5%
**Comorbidities**		
Diabetes	32,9%	39,1%
Kidney disease	8,8%	9,3%
Cardiac insufficiency	11,7%	12,0%
**Mortality rate**	2,6%	5,1%
**Cost**	8.397,3	23.116,4
**Scarcity**		
Urgent intervention	77,0%	38,2%
**Focus**		
Vacations	23,7%	15,1%
Rainy	15,9%	15,7%
*Number of discharges*	*357.860*	*47.058*

**Table 2:** Impact of scarcity and focus in percentage points of mortality

	PCI		CABG	
	Effect	P value	Effect	P value
Scarcity				
Urgent	1,50%	<0,0001	1,70%	<0,0001
Focus				
Vacations	0,11%	0,0367	0,21%	0,2614
Rainy day	-0,08%	0,1367	-0,68%	0,0201

Note: estimates for the Vacations effect are done including also the Urgent variable into the model, given that 84% of interventions in Vacations days are urgent, while for the non-vacations the percentage reduces to 67%. That is, we condition the effects to being in Vacations conditioned to being or not urgent, hence providing a robust estimate of the Vacation effect.

**Table 3:** Impact of scarcity and focus in euros of cost

	PCI		CABG	
	Effect	P value	Effect	P value
**Scarcity**				
Urgent	1.425,31	<0,0001	2.044,20	<0,0001
**Focus**				
Vacations	247,86	<0,0001	289,35	0,0321
Rainy day	-73,84	0,0055	-29,65	0,4219

Note: estimates for the Vacations effect are done including also the Urgent variable into the model, given that 84% of interventions in Vacations days are urgent, while for the non-vacations the percentage reduces to 67%

## Data Availability

The data used to support the findings of the present study are available from the corresponding authors upon request.

## References

[1] Zhao J, Tomm BM (2018). Psychological Responses to Scarcity. Oxford Res Encycl Psychol.

[2] Wulfemeyer J (2021). Cognitive Focus. Acta Anal.

[3] Spatt CS (2020). A Tale of Two Crises: The 2008 Mortgage Meltdown and the 2020 COVID-19 Crisis. Rev Asset Pricing Stud.

[4] Vo KD (2020). Evaluating the Role of Attention in Decision Making. Diss Duke Univ.

[5] Diamond A (2013). Executive Functions. https://doi.org/10.1146/annurev-psych-113011-143750.

[6] Posner MI (2014). Developing Attention: Behavioral and Brain Mechanisms. Adv Neurosci.

[7] Svarts A (2020). Does Focus Improve Performance in Elective Surgery? A Study of Obesity Surgery in Sweden. Int J Environ Res Public Health.

[8] David Zepeda E (2021). On the relations between focus, experience, and hospital performance. Health Care Manage Rev.

[9] Diwas Singh KC, Terwiesch C (2011). The Effects of Focus on Performance: Evidence from California Hospitals. https://doi.org/10.1287/mnsc.1110.1401.

[10] Bundhun PK, Bhurtu A, Yuan J (2017). Impact of type 2 diabetes mellitus on the long-term mortality in patients who were treated by coronary artery bypass surgery. Med (United States).

[11] Naidu SS (2003). Renal insufficiency is an independent predictor of mortality after percutaneous coronary intervention. Am J Cardiol.

[12] Garg S (2010). A new tool for the risk stratification of patients with complex coronary artery disease: the Clinical SYNTAX Score. Circ Cardiovasc Interv.

[13] Farooq V (2012). Combined anatomical and clinical factors for the long-term risk stratification of patients undergoing percutaneous coronary intervention: the Logistic Clinical SYNTAX score. Eur Heart J.

[14] Brendel F (2021). Resource scarcity and prioritization decisions in medical care: A lab experiment with heterogeneous patient types. Health Econ.

[15] Thomas M (2012). Does surgeon workload per day affect outcomes after pulmonary lobectomies?. Ann Thorac Surg.

[16] Hendey GW (2005). Overnight and postcall errors in medication orders. Acad Emerg Med.

[17] Landrigan CP (2004). Effect of reducing interns' work hours on serious medical errors in intensive care units. N Engl J Med.

[18] Lee JJ (2014). Rainmakers: why bad weather means good productivity. J Appl. Psychol.

[19] Connolly M (2008). Here Comes the Rain Again: Weather and the Intertemporal Substitution of Leisure. https://doi.org/10.1086/522067.

[20] Bryson A, Forth J Productivity and Days of the Week. 2007.

[21] Riggins FJ, Klamm BK (2017). Data governance case at KrauseMcMahon LLP in an era of self-service BI and Big Data. J Account Educ.

[22] Wenghofer EF (2009). Factors Affecting Physician Performance: Implications for Performance Improvement and Governance. Healthc Policy.

